# Survival prediction of glioblastoma patients—are we there yet? A systematic review of prognostic modeling for glioblastoma and its clinical potential

**DOI:** 10.1007/s10143-020-01430-z

**Published:** 2020-11-06

**Authors:** Ishaan Ashwini Tewarie, Joeky T. Senders, Stijn Kremer, Sharmila Devi, William B. Gormley, Omar Arnaout, Timothy R. Smith, Marike L. D. Broekman

**Affiliations:** 1 Department of Neurosurgery, Haaglanden Medical Center, Lijnbaan 32, 2512 VA The Hague, The Netherlands; 2 Faculty of Medicine, Erasmus University Rotterdam, Rotterdam, The Netherlands; 3 Computational Neurosciences Outcomes Center, Department of Neurosurgery, Brigham and Women’s Hospital, Harvard Medical School, Boston, MA USA; 4 King’s College, London, UK; 5 Department of Neurosurgery, Leiden University Medical Center, Leiden, The Netherlands

**Keywords:** Neurosurgery, Glioblastoma, Prognostic modeling, Overall survival

## Abstract

Glioblastoma is associated with a poor prognosis. Even though survival statistics are well-described at the population level, it remains challenging to predict the prognosis of an individual patient despite the increasing number of prognostic models. The aim of this study is to systematically review the literature on prognostic modeling in glioblastoma patients. A systematic literature search was performed to identify all relevant studies that developed a prognostic model for predicting overall survival in glioblastoma patients following the PRISMA guidelines. Participants, type of input, algorithm type, validation, and testing procedures were reviewed per prognostic model. Among 595 citations, 27 studies were included for qualitative review. The included studies developed and evaluated a total of 59 models, of which only seven were externally validated in a different patient cohort. The predictive performance among these studies varied widely according to the AUC (0.58–0.98), accuracy (0.69–0.98), and C-index (0.66–0.70). Three studies deployed their model as an online prediction tool, all of which were based on a statistical algorithm. The increasing performance of survival prediction models will aid personalized clinical decision-making in glioblastoma patients. The scientific realm is gravitating towards the use of machine learning models developed on high-dimensional data, often with promising results. However, none of these models has been implemented into clinical care. To facilitate the clinical implementation of high-performing survival prediction models, future efforts should focus on harmonizing data acquisition methods, improving model interpretability, and externally validating these models in multicentered, prospective fashion.

**Supplementary Information:**

The online version contains supplementary material available at 10.1007/s10143-020-01430-z.

## Introduction

Glioblastoma is the most common and aggressive type of primary brain tumor in adults [1–3]. It has one of the highest mortality rates among human tumors with a median survival of 12 to 15 months after diagnosis despite improved standard-of-care defined as maximal safe resection followed by radiotherapy plus concomitant and adjuvant temozolomide [1, 4].

Survival statistics are well-described at the population level, and many factors that impact survival have been identified including age, Karnofsky performance status (KPS), O6-methylguanine-DNA methyltransferase (MGMT) promotor methylation status, isocitrate dehydrogenase (IDH), neurological deficit, extent of resection, and tumor multifocality and location among others [5–7]. Yet, predicting individual patient survival remains challenging [7].

In recent years, prognostic models are increasingly being developed to predict survival of the individual glioblastoma patient [8, 9]. These prognostic models utilize a wide range of statistical and machine learning algorithms to analyze heterogenous data sources and predict individual patient survival. This systematic review aims to synthesize the current trends and provide an outlook concerning the possibility of clinical use of prognostic glioblastoma models and the future direction of survival modeling in glioblastoma patients.

## Methods

### Search strategy

A search was performed in the Embase, Medline Ovid (PubMed), Web of science, Cochrane CENTRAL, and Google Scholar databases according to the Preferred Reporting Items for Systematic Reviews and Meta-Analyses (PRISMA) guidelines (S1). A professional librarian was consulted for constructing the search syntax with the use of keywords for glioblastoma, prognostic modeling, and overall survival as well as their synonyms (S1). All prognostic models concerning survival in glioblastoma patients were included in our search syntax. Prediction model studies on glioblastoma patients with overall survival as the primary outcome were included. Predictor finding studies were excluded. These studies focus on characterizing the association between individual variables and the outcome at the cohort level (e.g., identifying risk factors of survival within a population), whereas prediction model studies seek to develop a model that predicts survival as accurately as possible in the individual patient utilizing the optimal combination of variables. No restrictions were applied with regard to the participant characteristics, format of the input data, type of algorithm, or validation of testing procedures. Case reports or articles written in languages other than Dutch or English were excluded. No restrictions based on the date of publication were used. This systematic search was complemented by screening the references of included articles to identify additional publications. Titles and abstracts of retrieved articles were screened by two independent authors. Two authors (IRT, SK) read the full texts of the potentially eligible articles independently. Discrepancies were solved by discussion including a third reviewer (JS).

### Data extraction

From all included studies, we extracted the year of publication, name of first author, title, abstract, source of data, selection criteria, events per variable, events, sample size, type of input, hyperparameter tuning, number of predictors in the best performing model, definition of overall survival, algorithm type, validation and testing procedure, performance metric, and model performance. To ensure a systematic approach of assessing validation of prognostic modeling in glioblastoma, all the extracted variables were based on the CHecklist for critical Appraisal and data extraction for systematic Reviews of prediction Modelling Studies (CHARMS) checklist [10]. The Prediction model Risk Of Bias ASsessment Tool (PROBAST) tool was used to assess the risk of bias in all included studies [11].

## Results

**Fig. 1:**
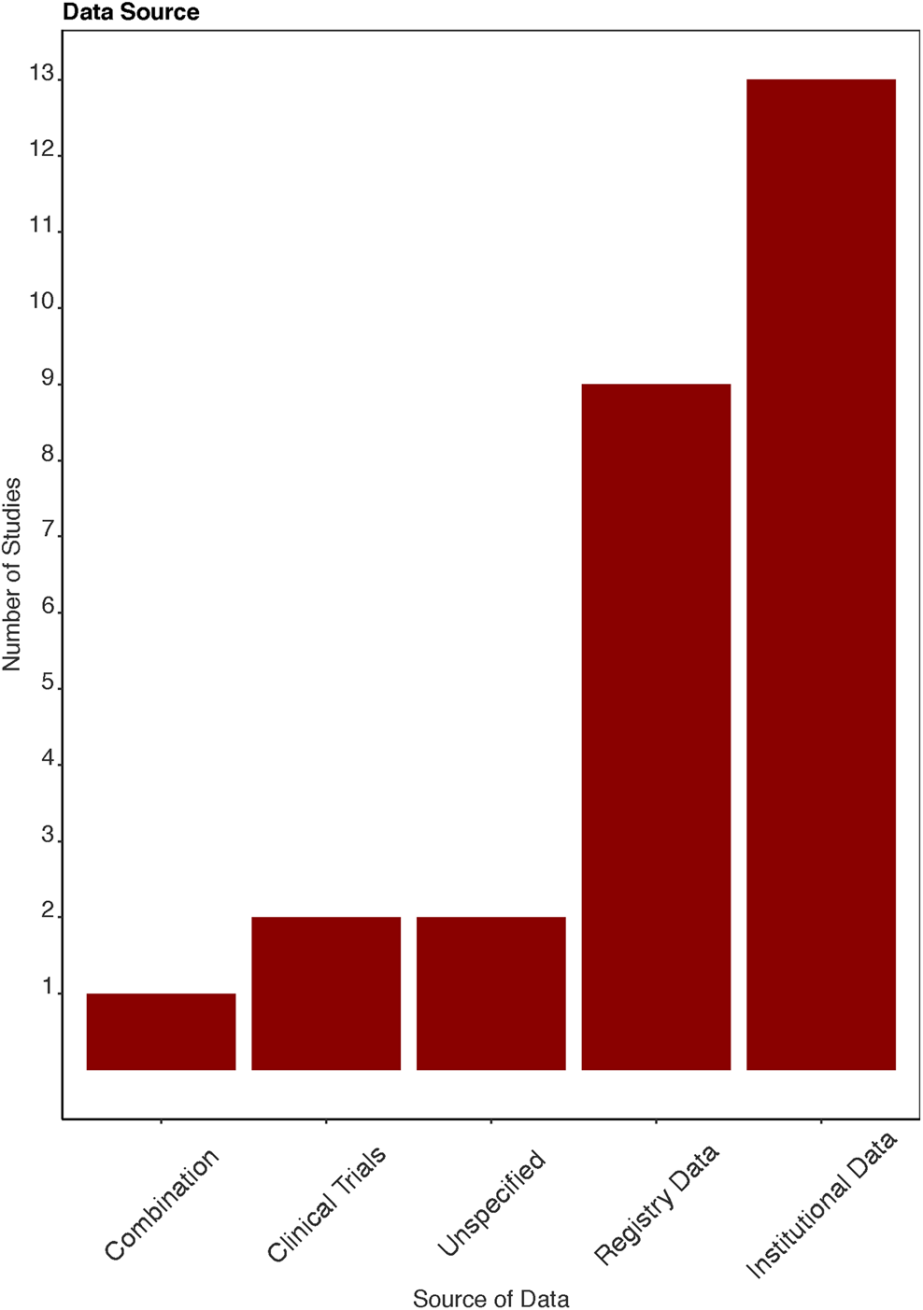
Data source

**Fig. 2:**
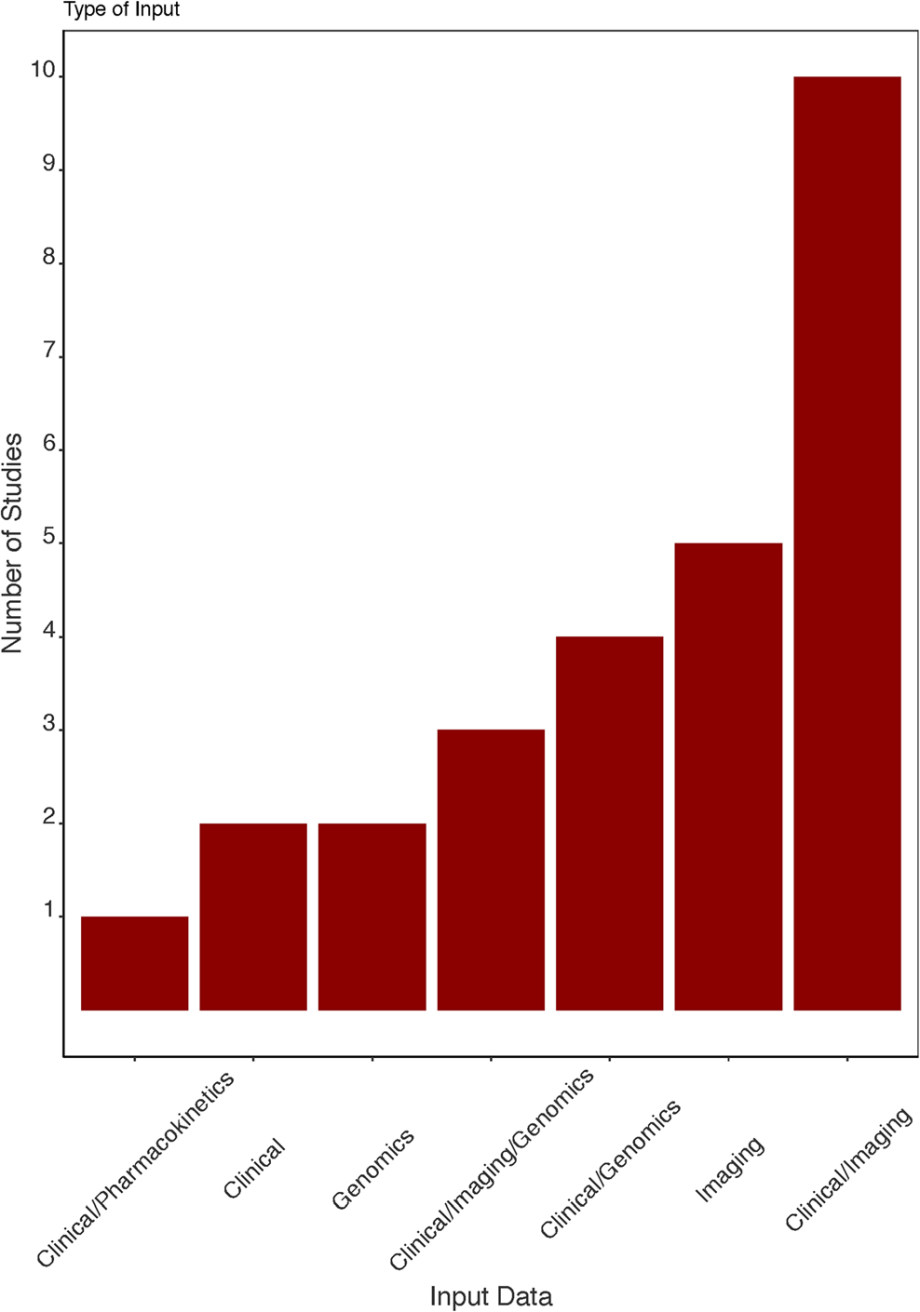
Type of input

**Fig. 3:**
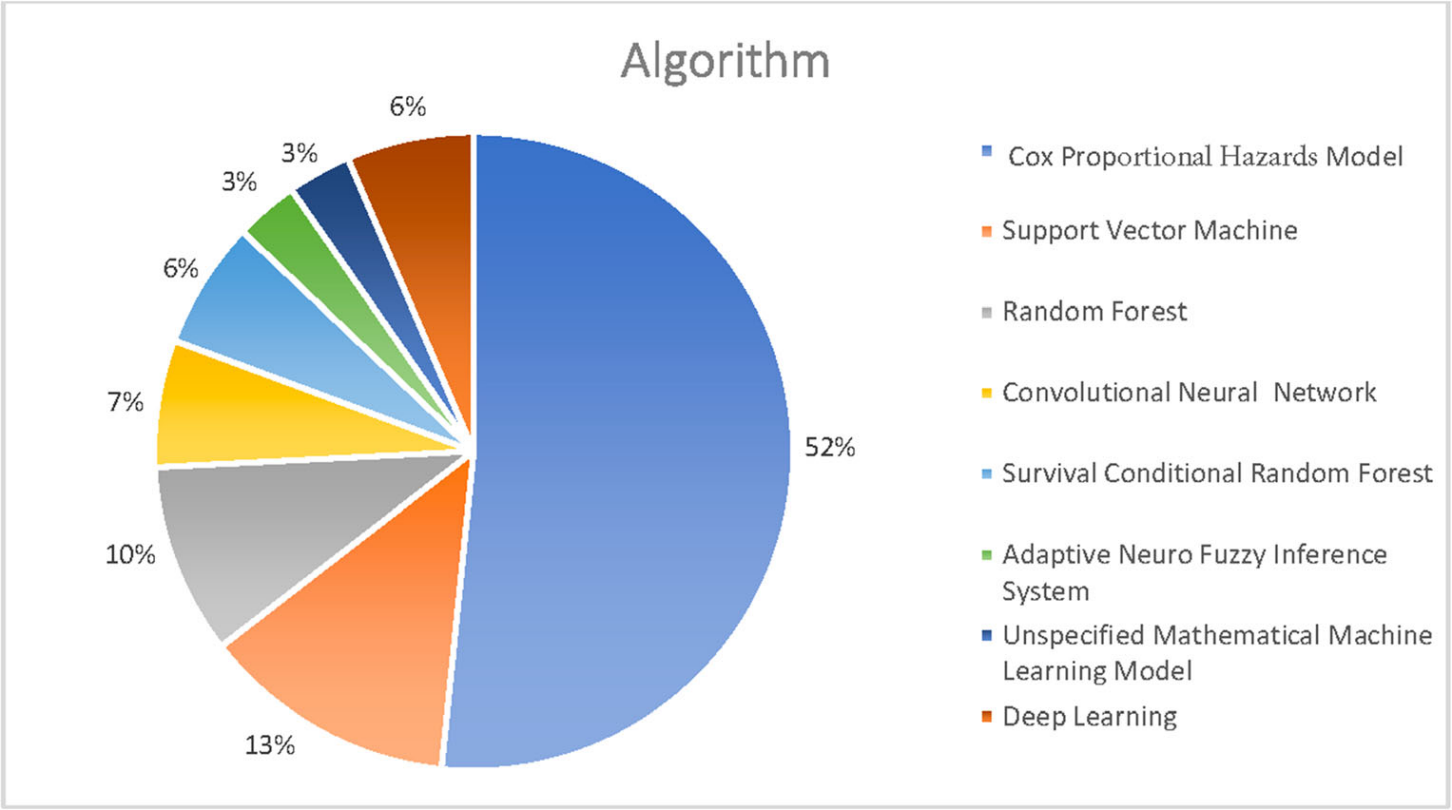
Model characteristics: algorithm type and validation strategy across all identified glioblastoma models

The search identified 595 unique studies. After screening by title and abstract and subsequently screening the full text, 112 studies were included for full-text review (S2). Of these, 27 articles met our inclusion criteria and were included in the qualitative synthesis [12–38]. A total of 59 models were presented of which the best performing model was included in this review. Two included models used the same database [14, 36]. Yet, both were included in this systematic review because different predictors and algorithms were used to develop the models. The included prognostic models were developed between 2010 and 2019. General characteristics and model characteristics for each included study are presented in Supplementary S3. An overview of observations in all identified glioblastoma prognostic models is visualized in Figs. 1, 2, and 3.

### Participants

The data that were used to develop glioblastoma survival prediction models were retrieved from clinical trials (*n* = 2) [26, 30], institutional data (*n* = 13) [13–15, 19, 20, 22, 25, 28, 29, 32–34, 36], registry data (*n* = 9) [12, 16, 18, 21, 23, 24, 27, 31, 38], combined institutional and database data (*n* = 1) [35], and unspecified data sources (*n* = 2) [17, 37]. Twelve models used data from consecutive patients [12, 13, 15, 23–29, 35, 38]. Fifteen models did not explicitly specify the selection criteria or procedures [12, 14, 16, 17, 21–24, 26, 27, 31, 33, 35–37] (Fig. 1).

### Type of input

The utilized data sources were clinical parameters (*n* = 2) [19, 32]; genomics (*n* = 2) [12, 21]; MRI imaging (*n* = 4) [22, 23, 34, 37]; combined clinical and genomics (*n* = 4) [13, 16, 27, 28]; combined clinical and MRI imaging (*n* = 10) [14, 18, 20, 24, 25, 29–31, 35, 38]; combined clinical, MRI imaging, and genomics (*n* = 3) [15, 26, 36]; histopathology (*n* = 1) [33]; and combined clinical and pharmacokinetics (*n* = 1) [17]. Up to 2017, only two studies analyzed high-dimensional data sources (i.e., MRI or genomic information) in addition to clinical information [12, 13]. From 2017 onwards, there was a substantial increase in the use of genomic [15, 16, 21, 26–28, 36] and imaging [15, 26, 36, 38] data for survival modeling in glioblastoma patients (Fig. 2).

### Algorithm type

Various statistical and machine learning algorithms were used to predict survival in glioblastoma patients including Cox proportional hazards regression (*n* = 16) [11–13, 16, 19, 20, 24–28, 30, 33, 35, 36, 38], support vector machine (*n* = 4) [22, 31, 37, 38], random forest (*n* = 3) [14, 29, 38], convolutional neural networks (*n* = 2) [18, 23]), adaptive neuro-fuzzy inference system (*n* = 1) [17], and an unspecified mathematical machine learning model (*n* = 1) [34]. To date, only two deep learning models (i.e., convolutional neural networks) have been developed for predicting survival in glioblastoma patients [18, 23]. Although classical statistical algorithms are still being used for model development, a rapid increase in the use of machine learning can be seen from 2016 onwards (Fig. 3).

### Outcome definition

Overall survival was modeled as a continuous (*n* = 7) [15, 17, 26, 28, 30, 35, 38], binary (*n* = 11) [16, 19, 22, 24, 25, 27, 29, 31, 34, 37, 38], or time-to-event outcome (*n* = 11) [12–14, 18, 20, 21, 23, 32, 33, 36, 38]. In studies that defined survival as a binary outcome, survival was dichotomized into short and long survival at 6 [19], 12 [27, 38], and 18 months [37], more or less than 400 days [31], as well as the median [29] and mean [25] overall survival in the training cohort.

### Validation and testing procedures

Hyperparameter settings were optimized using various validation strategies. Thirteen models divided the original dataset into separate training and validation sets [12, 14–16, 19, 20, 23, 27–29, 32, 35, 36]. Twelve studies applied a cross-validation strategy including leave one out (*n* = 4) [22, 24, 25, 34], 5-fold (*n* = 3) [13, 31, 38], 10-fold (*n* = 2) [18, 26], 33-fold (*n* = 1) [17], leave 3 out (*n* = 1) [37], and unspecified cross-validation (*n* = 1) [21]. Bootstrap validation was mentioned in three studies [26, 30, 36]. Two studies used the same subset for validation and testing [27, 33]. Most studies used a separate, prefixed subset of the original data as the hold-out test set to avoid overfitting on the validation set. Seven studies even evaluated model performance on patients from a different data source, thereby developing a model on patients from one institution and testing on patients from another [14–16, 18, 23, 26, 32].

### Performance metrics and performance

**Fig. 4:**
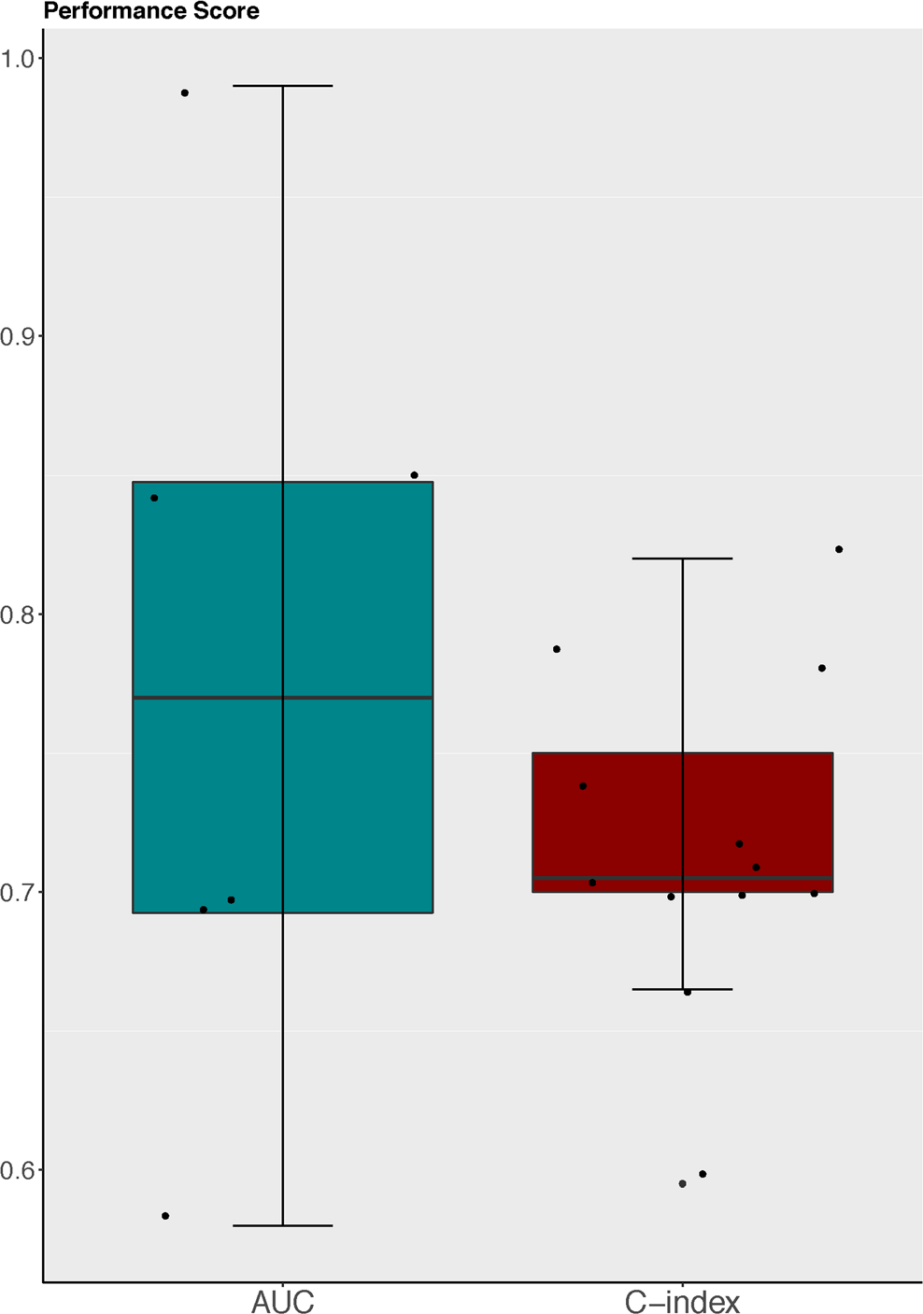
Performance score

The performance of a prognostic model can be expressed according to various statistics depending on the type of outcome format used in the model. In prognostic models, discrimination and calibration are among the most commonly used metrics for measuring model performance [39, 40]. Discrimination is the ability to distinguish cases from non-cases and is often expressed as the area under the receiver operating characteristic curve (AUC) [41] or Harrel’s C-index [42, 43]. Harrel’s C-index is an extension of the AUC considering the occurrence of the event, as well as the length of follow-up, thereby particularly well-suited for right-censored survival data [42]. Calibration constitutes the agreement between the observed and predicted outcomes and is often graphically expressed as a calibration plot or numerically as the calibration slope and intercept [41]. Definitions of other performance metrics can be found in Table 1 [41, 42]. Ten models [16, 19, 22, 24, 25, 27, 29, 31, 34, 37] with a binary outcome format expressed performance as the AUC ranging between 0.58 [19] and 0.98 [16] (*n* = 7) (Fig. 4) [16, 17, 19, 24, 27, 30, 37], accuracy ranging from 0.69 [29] to 0.98 [31] (*n* = 5) [22, 25, 29, 31, 37], and a calibration curve (*n* = 1) [25]. Studies modeling survival as a continuous outcome (*n* = 7) [15, 17, 26, 28, 30, 35, 38] measured the prediction performance using Harrell’s C-statistic which ranged between 0.66 [26] and 0.70 [15, 28] (*n* = 3) [15, 26, 28], and a calibration plot (*n* = 2) [26, 38]. The eleven time-to-event models [12–14, 18, 20, 21, 23, 32, 33, 36, 38] applied the C-index ranging between 0.70 [12, 14, 32, 38] and 0.82 [13] (Fig. 4) (*n* = 10) [12–14, 18, 20, 21, 23, 32, 36, 38], and one model [33] did not describe the model performance [41–43] (Table 1).

**Table 1:** Definition of performance metrics

Performance metric	Definition
Receiver operating characteristics curve (ROC)	A probability curve of true-positive rates against the false-positive rates at different cutoff points in outcome [1].
Area under the ROC curve	Area under the curve (AUC) distinguishes the discriminative potential of the algorithm. The threshold is 0.5 (no discriminative ability) [41].
Harrell’s C-statistic	To quantify the estimation of the algorithm in discriminating among subjects with different time events. Harrell’s approach utilizes a normalizing constant for right-censored data [1–3].
Calibration curve	The agreement between the predictions (*x*-axis) and observed outcome (*y*-axis) [1–3].
Concordance index	To quantify the estimation of the algorithm in discriminating among subjects with different time events. Utilizes a normalizing constant for left censored data [1–3].

### Online prediction tools

Three studies have translated their model into an online prediction tool making the models more actionable and useful for individual prognostication in glioblastoma patients [26, 30, 38]. Although these three models included radiographic features, such as tumor size and extension, these features have to be interpreted or measured manually by a human expert and inserted into the model. Therefore, none of the online prediction tools used raw MRI data but exclusively used structured clinical parameters. Although studies have developed deep learning models utilizing unstructured data, e.g., genomics [26] or MRI imaging [38], none of these models has been translated to an actionable clinical prediction tool yet.

### Risk of bias

Using the PROBAST tool [11] and CHARMS guidelines [10], risk of bias was assessed. This showed that three models had a potentially high risk of bias [14, 16, 33]. Seven models had an intermediate risk [13, 21, 22, 26, 29, 31, 35] with 17 of all models being low risk. The performed assessment of included models found a risk of bias in the following domains: participants (*n* = 4) [17, 26, 31, 37], predictors (*n* = 10) [12–14, 16, 20–22, 26, 29, 33], outcomes (*n* = 7) [13, 14, 16, 21, 22, 29, 33], and analysis (*n* = 7) [14, 16, 25, 31, 33, 35, 36]. When using manually measured imaging parameters, ambiguity in assessment can occur; there was no form of double-blinded assessment of MRI images which prevents objectivity. Additionally, models did not define how some parameters, such as extent of resection, were measured. Some models also seem to overfit in analysis on existing data with manners as a skewed divide of training/testing dataset or a low events per variable and not accounting for missing data. Fourteen models did not include all initially included patients in analysis of the best performing model [12–15, 19, 21, 25, 27–30, 32, 34, 36]. Out of 14 models without complete enrolment of patients, 7 models did not describe the type and/or frequency of missing data [12, 15, 19, 21, 25, 29, 34] (Table 2) (S4).

**Table 2:** ROB assessment included models

Study	ROB				Overall
	Participants	Predictors	Outcome	Analysis	
Youssef et al. [12]	+	−	+	+	+
Michaelsen et al. [13]	+	−	−	+	?
Peeken et al. [14]	+	−	−	−	−
Woo et al. [15]	+	+	+	+	+
Liang et al. [16]	+	−	−	−	−
Dehkordi et al. [17]	−	+	+	+	+
Lao et al. [18]	+	+	+	+	+
Urup et al. [19]	+	+	+	+	+
Park et al. [20]	+	−	+	+	+
Xia et al. [21]	+	−	−	+	?
Upadhaya et al. [22]	+	−	−	+	?
Li et al. [23]	+	+	+	+	+
Mazurowski et al. [24]	+	+	+	+	+
Fuster-Garcia et al. [25]	+	+	+	−	+
Gittleman et al. [26]	−	−	+	+	?
Ai et al. [27]	+	+	+	+	+
Molitoris et al. [28]	+	+	+	+	+
Chang et al. [29]	+	−	−	+	?
Gorlia et al. [30]	+	+	+	+	+
Sanghani et al. [31]	−	+	+	−	?
Audureau et al. [32]	+	+	+	+	+
Yuan et al. [33]	+	−	−	−	−
Neal et al. [34]	+	+	+	+	+
Park et al. [35]	+	+	+	−	?
Peeken et al. [36]	+	+	+	+	+
Zacharaki et al. [37]	−	+	+	+	+

Adapted from PROBAST (Prediction model Risk Of Bias ASsessment Tool); *ROB* risk of bias

“+” indicates low ROB; “−” indicates high ROB; “?” indicates unclear ROB

## Discussion

This systematic review demonstrates the lack of widespread validation and clinical use of the existing glioblastoma models. Despite the increasing development of survival prediction models for glioblastoma patients, only seven model have been validated retrospectively in an external patient cohort [14–16, 18, 23, 26, 32], and none has been validated prospectively. Furthermore, three models, all of which developed from a statistical algorithm, have been deployed as a publicly available prediction tool [26, 30, 38], but none has been implemented as a standardized tool to guide clinical decision-making. Lastly, no trend was seen in performance throughout time despite machine learning methods increasingly being used, and no prognostic glioblastoma study till date had consequences for clinical decision-making.

Prognostic models have the potential to help tailor clinical management to needs of the individual glioblastoma patient by providing a personalized risk-benefit analysis. The increase of machine learning algorithms, and deep learning, enables the use of high-dimensional data, such as free text and imaging, to improve the accuracy and performance of prediction models. The increasing use of machine learning for the analysis of unstructured, high-dimensional data parallels the current trends in predictive modeling in medicine [44, 45]. Neurosurgical examples include machine learning algorithms for glioblastoma, deep brain stimulation, traumatic brain injury, stroke, and spine surgery [38, 44–53]. Deep learning algorithms are also increasingly being used to further improve the WHO 2016 classification of high-grade gliomas via histological and biomolecular variables for more concise diagnosis and classification of gliomas [54–56].

Furthermore, deep learning algorithms are also frequently used in radiotherapeutic research for automated skull stripping, automated segmentation, or delineation of resection cavities for stereotactic radiosurgery [57–60]. Despite the ubiquity of high-performing models in clinical research, none has been translated to the clinical realm and integrated in clinical decision-making. Few prognostic models are put to practice throughout all medical specialties. Specifically, for other diseases, such as breast cancer, where radiological and genetic factors are more well-known than glioblastoma, more than sixty models were found to prognosticate breast cancer survival [61]. Nevertheless, this has led to little consequence for clinical care [62]. Seemingly, clinical implementation is not a matter of robustness of evidence or notoriety of the disease. Moons et al. also parallel our finding that robust validation studies are missing for most prognostic models and that most validation studies include a relatively small patient cohort, thus not helping the model’s generalizability [62]. This raises the question: what needs to happen before prognostic models can be used in clinic?

### Computational challenges

First, machine learning algorithms are accompanied with unique computational challenges which could limit the clinical implementation of these models. Due to the high-dimensionality of the input data, machine learning models are inherently limited in their generalizability. Computer vision models developed on single institutional data perform poorly on data from different institutions when different scanning parameters, image features, and other formatting methods are used [63]. In contrast, prediction models that exclusively use structured clinical parameters, such as age, gender, and the presence of comorbidity, are more generalizable and implementable across institutions as this information is less subject to institution-specific data acquisition methods [26, 30, 38]. This could be one of the reasons that the three currently available prediction models all exclusively use clinically structured information. Therefore, if unstructured data and machine learning algorithms are to be incorporated in clinical prognostic models at multicenter level, harmonization and standardization procedures are required between institutions.

Furthermore, most machine learning algorithms accommodate primarily to binary or continuous outcomes, whereas survival data is typically composed of right-censored data, in which the value of an observation is only partially known (i.e., the patient survived at least beyond a specific follow-up time). Other approaches have already been considered for handling aforementioned data, such as discarding, including it twice in the model: once as an event and once as event-free, which creates bias in risk estimate. In addition, novel approaches such as modifying specific machine learning algorithms or weight estimation of the amount of censoring in sample size are introduced [64–66]. This highlights the need for translating existing machine learning algorithms to alternatives that can accommodate time-to-event survival data as well.

### Clinical challenges

Machine learning algorithms are also accompanied with unique clinical challenges which could limit their clinical implementation. As medical computational science progresses, critical unanswered questions arise: (to what extent) should a medical professional rely on technology and how do you intercept an inevitable predictive miscalculation of the algorithm? First, the black box of many algorithms, e.g., hidden layers in neural networks, substantially reduces the interpretability of a potentially high-performing prediction model and thereby limits their clinical deployment. However, this is not a new phenomenon: therapeutic measures can be implemented in clinical care based on studies demonstrating their safety and efficacy, yet without the underlying mechanisms being fully clarified. In addition, algorithms learn from real-world data, and therefore, real-world disparities could propagate into the developed models. This could potentially sustain or amplify existing healthcare disparities, such as ethnic or racial biases [67]. If survival prediction models were to be clinically deployed, should these algorithms be used as a directive or supportive application in clinical decision-making? There is insufficient information and experience up until now to fully answer this question. Liability can become an issue if a misprediction, e.g., false positive, is made and clinical decisions are influenced by this misprediction; is the clinician responsible for the algorithms’ fault? Therefore, medical professionals should be cautious when relying on technology and attempt to further understand predictive algorithms and their inevitable limitations.

### Standardizing model evaluation prior to clinical implementation

These computational and clinical challenges have led to the development standardized methods of assessing predictive models for clinical implementation, both in the diagnostic and prognostic realms. The use of methylome data in neuro-oncology [68] has the promise to be used clinically as recommended by the iMPACT-now guidelines [69]. This study performed a prospective external validation in five different centers to test the accuracy of their model. Additionally, the model was tested in two different labs for technical robustness. This could be the most important step towards clinical deployment of prognostic glioblastoma models as well. Moreover, prognostic radiomics models for GBM patients demonstrate significant potential to achieve noninvasive pathological diagnosis and prognostic stratification of glioblastoma [18, 23]. Yet, technical challenges need to be overcome before implementation of these models is realized, namely the access to larger image datasets, common criteria for feature definitions and image acquisition, and wide-scale validation of one radiomics model [70]. Another neuro-oncological model that is widely used for research purposes is the ds-GPA that functions as a diagnostic prognosis assessment for brain metastases [71]. Sperduto et al. included shortcomings of the ds-GPA, but more importantly report possible consequences in clinical decision-making per prognostic score of the ds-GPA [71]. This offers clinicians insight in the utility of the ds-GPA. The caveat of prognostic glioblastoma models is the lack of appraisal concerning the collected clinical data. Moreover, the FDA considers appraisal of data and subsequent analysis of all gathered data as a preliminary for clinical deployment of software as a medical device. Concisely, iterated datasets that could be considered as “pivotal” for superior performance, safety, and specific risk definitions should be identified before clinical deployment or introduction into guidelines [72].

### Limitations

There are several limitations to the current systematic review. First, a preferred quantitative meta-analysis was not possible due to the methodological heterogeneity across all studies, and differences in model performance should be interpreted with caution. As low-performing models are not published, common bottlenecks may remain unexposed resulting in a duplication of futile efforts. Furthermore, publication bias can influence results as previously mentioned; high-performing models are likelier to be published. To the best of our knowledge, there are no previous systematic reviews presenting a general overview of the emerging field of survival modeling in glioblastoma patients.

### Future directions

As the current field of survival modeling in glioblastoma patients is gravitating towards high-dimensional models, future research efforts should focus on harmonization and standardization to increase the volume of available training data, the accuracy of developed models, and the generalizability of their associated prediction tools. As of now, models specifically report prediction performance, yet there are many secondary characteristics that determine whether or not a model can be implemented in clinical practice. Therefore, future studies should concentrate not only on model performance but also on secondary metrics, such as the interpretability and ease of use, that are relevant for their clinical deployment [38]. Moreover, future research should focus on clinical utility, i.e., explaining how or when clinicians should alter the treatment plan of the glioblastoma patient. Lastly, considering the ethical and clinical implications parallel to its development could ensure a safe and sound implementation of this rapidly emerging technology.

## Conclusion

The use of machine learning algorithms in prognostic survival models for glioblastoma has increased progressively in recent years. Yet, no machine learning models have led to an actionable prediction tool to date. For successful translation of a tool to the clinic, multicentered standardization and harmonization of data are needed. Future studies should focus not only on the model performance, but also on the secondary model characteristics, such as interpretability and ease of use, and the ethical challenges accompanied with it to ensure a safe and effective implementation in clinical care.

## Supplementary Information

ESM 1 (DOCX 54 kb).

## References

[1] Wang Y, Liu X, Guan G, Zhao W, Zhuang M (2019). A risk classification system with five-gene for survival prediction of glioblastoma patients. Front Neurol.

[2] Omuro A, DeAngelis LM (2013). Glioblastoma and other malignant gliomas: a clinical review. Jama.

[3] Kucharczyk MJ, Parpia S, Whitton A, Greenspoon JN (2017). Evaluation of pseudoprogression in patients with glioblastoma. Neurooncol Pract.

[4] Wen PY, Kesari S (2008). Malignant gliomas in adults. N Engl J Med.

[5] Lutterbach J, Sauerbrei W, Guttenberger R (2003). Multivariate analysis of prognostic factors in patients with glioblastoma. Strahlenther Onkol.

[6] Stark AM, van de Bergh J, Hedderich J, Mehdorn HM, Nabavi A (2012). Glioblastoma: clinical characteristics, prognostic factors and survival in 492 patients. Clin Neurol Neurosurg.

[7] Zhao YH, Wang ZF, Pan ZY, Peus D, Delgado-Fernandez J, Pallud J, Li ZQ (2019). A meta-analysis of survival outcomes following reoperation in recurrent glioblastoma: time to consider the timing of reoperation. Front Neurol.

[8] Molina-García D, Vera-Ramírez L, Pérez-Beteta J, Arana E, Pérez-García VM (2019). Prognostic models based on imaging findings in glioblastoma: human versus machine. Sci Rep.

[9] Narang S, Lehrer M, Yang D, Lee J, Rao A (2016). Radiomics in glioblastoma: current status, challenges and potential opportunities. J Transl Cancer Res.

[10] Moons KG, de Groot JA, Bouwmeester W, Vergouwe Y, Mallett S, Altman DG, Reitsma JB, Collins GS (2014). Critical appraisal and data extraction for systematic reviews of prediction modelling studies: the CHARMS checklist. PLoS Med.

[11] Wolff RF, Moons KGM, Riley RD, Whiting PF, Westwood M, Collins GS, Reitsma JB, Kleijnen J, Mallett S (2019). PROBAST: a tool to assess the risk of bias and applicability of prediction model studies. Ann Intern Med.

[12] Youssef I, Clarke R, Shih Ie M, Wang Y, Yu G (2016). Biologically inspired survival analysis based on integrating gene expression as mediator with genomic variants. Comput Biol Med.

[13] Michaelsen SR, Christensen IJ, Grunnet K, Stockhausen MT, Broholm H, Kosteljanetz M, Poulsen HS (2013). Clinical variables serve as prognostic factors in a model for survival from glioblastoma multiforme: an observational study of a cohort of consecutive non-selected patients from a single institution. BMC Cancer.

[14] Peeken JC, Goldberg T, Pyka T, Bernhofer M, Wiestler B, Kessel KA, Tafti PD, Nusslin F, Braun AE, Zimmer C, Rost B, Combs SE (2019). Combining multimodal imaging and treatment features improves machine learning-based prognostic assessment in patients with glioblastoma multiforme. Cancer Med.

[15] Woo P, Ho J, Lam S, Ma E, Chan D, Wong WK, Mak C, Lee M, Wong ST, Chan KY, Poon WS (2018). A comparative analysis of the usefulness of survival prediction models for patients with glioblastoma in the temozolomide era: the importance of methylguanine methyltransferase promoter methylation, extent of resection, and subventricular zone location. World Neurosurg.

[16] Liang R, Wang M, Zheng G, Zhu H, Zhi Y, Sun Z (2018). A comprehensive analysis of prognosis prediction models based on pathway-level, gene-level and clinical information for glioblastoma. Int J Mol Med.

[17] Dehkordi ANV, Kamali-Asl A, Wen N, Mikkelsen T, Chetty IJ, Bagher-Ebadian H (2017) DCE-MRI prediction of survival time for patients with glioblastoma multiforme: using an adaptive neuro-fuzzy-based model and nested model selection technique. NMR Biomed 30. 10.1002/nbm.3739 10.1002/nbm.3739 28543885

[18] Lao J, Chen Y, Li ZC, Li Q, Zhang J, Liu J, Zhai G (2017). A deep learning-based radiomics model for prediction of survival in glioblastoma multiforme. Sci Rep.

[19] Urup T, Dahlrot RH, Grunnet K, Christensen IJ, Michaelsen SR, Toft A, Larsen VA, Broholm H, Kosteljanetz M, Hansen S, Poulsen HS, Lassen U (2016). Development and validation of a prognostic model for recurrent glioblastoma patients treated with bevacizumab and irinotecan. Acta Oncol.

[20] Park M, Lee SK, Chang JH, Kang SG, Kim EH, Kim SH, Song MK, Ma BG, Ahn SS (2017). Elderly patients with newly diagnosed glioblastoma: can preoperative imaging descriptors improve the predictive power of a survival model?. J Neuro-Oncol.

[21] Xia Y, Yang C, Hu N, Yang Z, He X, Li T, Zhang L (2017). Exploring the key genes and signaling transduction pathways related to the survival time of glioblastoma multiforme patients by a novel survival analysis model. BMC Genomics.

[22] Upadhaya T, Morvan Y, Stindel E, Le Reste PJ, Hatt M (2015). A framework for multimodal imaging-based prognostic model building: preliminary study on multimodal MRI in glioblastoma multiforme. Irbm.

[23] Li Q, Bai H, Chen Y, Sun Q, Liu L, Zhou S, Wang G, Liang C, Li ZC (2017). A fully-automatic multiparametric radiomics model: towards reproducible and prognostic imaging signature for prediction of overall survival in glioblastoma multiforme. Sci Rep.

[24] Mazurowski MA, Desjardins A, Malof JM (2013). Imaging descriptors improve the predictive power of survival models for glioblastoma patients. Neuro-Oncology.

[25] Fuster-Garcia E, Juan-Albarracin J, Garcia-Ferrando GA, Marti-Bonmati L, Aparici-Robles F, Garcia-Gomez JM (2018). Improving the estimation of prognosis for glioblastoma patients by MR based hemodynamic tissue signatures. NMR Biomed.

[26] Gittleman H, Lim D, Kattan MW, Chakravarti A, Gilbert MR, Lassman AB, Lo SS, Machtay M, Sloan AE, Sulman EP, Tian D, Vogelbaum MA, Wang TJC, Penas-Prado M, Youssef E, Blumenthal DT, Zhang P, Mehta MP, Barnholtz-Sloan JS (2017). An independently validated nomogram for individualized estimation of survival among patients with newly diagnosed glioblastoma: NRG Oncology RTOG 0525 and 0825. Neuro-Oncology.

[27] Ai Z, Li L, Fu R, Lu JM, He JD, Li S (2017). Integrated Cox’s model for predicting survival time of glioblastoma multiforme. Tumour Biol.

[28] Molitoris JK, Rao YJ, Patel RA, Kane LT, Badiyan SN, Gittleman H, Barnholtz-Sloan JS, Bentzen SM, Kruser TJ, Huang J, Mehta MP (2017). Multi-institutional external validation of a novel glioblastoma prognostic nomogram incorporating MGMT methylation. J Neuro-Oncol.

[29] Chang K, Zhang B, Guo X, Zong M, Rahman R, Sanchez D, Winder N, Reardon DA, Zhao B, Wen PY, Huang RY (2016). Multimodal imaging patterns predict survival in recurrent glioblastoma patients treated with bevacizumab. Neuro-Oncology.

[30] Gorlia T, Stupp R, Brandes AA, Rampling RR, Fumoleau P, Dittrich C, Campone MM, Twelves CC, Raymond E, Hegi ME, Lacombe D, van den Bent MJ (2012). New prognostic factors and calculators for outcome prediction in patients with recurrent glioblastoma: a pooled analysis of EORTC Brain Tumour Group phase I and II clinical trials. Eur J Cancer.

[31] Sanghani P, Ang BT, King NKK, Ren H (2018). Overall survival prediction in glioblastoma multiforme patients from volumetric, shape and texture features using machine learning. Surg Oncol.

[32] Audureau E, Chivet A, Ursu R, Corns R, Metellus P, Noel G, Zouaoui S, Guyotat J, Le Reste PJ, Faillot T, Litre F, Desse N, Petit A, Emery E, Lechapt-Zalcman E, Peltier J, Duntze J, Dezamis E, Voirin J, Menei P, Caire F, Dam Hieu P, Barat JL, Langlois O, Vignes JR, Fabbro-Peray P, Riondel A, Sorbets E, Zanello M, Roux A, Carpentier A, Bauchet L, Pallud J, Club de Neuro-Oncologie of the Societe Francaise de N (2018). Prognostic factors for survival in adult patients with recurrent glioblastoma: a decision-tree-based model. J Neuro-Oncol.

[33] Yuan JX, Bafakih FF, Mandell JW, Horton BJ, Munson JM (2016). Quantitative analysis of the cellular microenvironment of glioblastoma to develop predictive statistical models of overall survival. J Neuropathol Exp Neurol.

[34] Neal ML, Trister AD, Ahn S, Baldock A, Bridge CA, Guyman L, Lange J, Sodt R, Cloke T, Lai A, Cloughesy TF, Mrugala MM, Rockhill JK, Rockne RC, Swanson KR (2013). Response classification based on a minimal model of glioblastoma growth is prognostic for clinical outcomes and distinguishes progression from pseudoprogression. Cancer Res.

[35] Park JK, Hodges T, Arko L, Shen M, Dello Iacono D, McNabb A, Olsen Bailey N, Kreisl TN, Iwamoto FM, Sul J, Auh S, Park GE, Fine HA, Black PM (2010). Scale to predict survival after surgery for recurrent glioblastoma multiforme. J Clin Oncol.

[36] Peeken JC, Hesse J, Haller B, Kessel KA, Nusslin F, Combs SE (2018). Semantic imaging features predict disease progression and survival in glioblastoma multiforme patients. Strahlenther Onkol.

[37] Zacharaki EI, Morita N, Bhatt P, O'Rourke DM, Melhem ER, Davatzikos C (2012). Survival analysis of patients with high-grade gliomas based on data mining of imaging variables. AJNR Am J Neuroradiol.

[38] Senders JT, Staples P, Mehrtash A, Cote DJ, Taphoorn MJB, Reardon DA, Gormley WB, Smith TR, Broekman ML, Arnaout O (2019). An online calculator for the prediction of survival in glioblastoma patients using classical statistics and machine learning. Neurosurgery.

[39] Mallett S, Royston P, Waters R, Dutton S, Altman DG (2010) Reporting performance of prognostic models in cancer: a review. 8:21. 10.1186/1741-7015-8-21 10.1186/1741-7015-8-21 PMC2857810 20353579

[40] Pencina MJ, D'Agostino RB, D'Agostino RB, Vasan RS (2008). Evaluating the added predictive ability of a new marker: from area under the ROC curve to reclassification and beyond. Stat Med.

[41] Steyerberg EW, Vickers AJ, Cook NR, Gerds T, Gonen M, Obuchowski N, Pencina MJ, Kattan MW (2010). Assessing the performance of prediction models: a framework for traditional and novel measures. Epidemiology.

[42] Uno H, Cai T, Pencina MJ, D'Agostino RB, Wei LJ (2011). On the C-statistics for evaluating overall adequacy of risk prediction procedures with censored survival data. Stat Med.

[43] Brentnall AR, Cuzick J (2018). Use of the concordance index for predictors of censored survival data. Stat Methods Med Res.

[44] Senders JT, Staples PC, Karhade AV, Zaki MM, Gormley WB, Broekman MLD, Smith TR, Arnaout O (2018). Machine learning and neurosurgical outcome prediction: a systematic review. World Neurosurg.

[45] Celtikci E (2018). A systematic review on machine learning in neurosurgery: the future of decision-making in patient care. Turk Neurosurg.

[46] Baumgarten C, Haegelen C, Zhao Y, Sauleau P, Jannin P (2018). Data-driven prediction of the therapeutic window during subthalamic deep brain stimulation surgery. Stereotact Funct Neurosurg.

[47] Donald R, Howells T, Piper I, Enblad P, Nilsson P, Chambers I, Gregson B, Citerio G, Kiening K, Neumann J, Ragauskas A, Sahuquillo J, Sinnott R, Stell A (2019). Forewarning of hypotensive events using a Bayesian artificial neural network in neurocritical care. J Clin Monit Comput.

[48] Hale AT, Stonko DP, Brown A, Lim J, Voce DJ, Gannon SR, Le TM, Shannon CN (2018). Machine-learning analysis outperforms conventional statistical models and CT classification systems in predicting 6-month outcomes in pediatric patients sustaining traumatic brain injury. Neurosurg Focus.

[49] Hu B, Kim C, Ning X, Xu X (2018). Using a deep learning network to recognise low back pain in static standing. Ergonomics.

[50] Liu J, Chen Y, Lan L, Lin B, Chen W, Wang M, Li R, Yang Y, Zhao B, Hu Z, Duan Y (2018). Prediction of rupture risk in anterior communicating artery aneurysms with a feed-forward artificial neural network. Eur Radiol.

[51] Popovic M, Lemke M, Zeng L, Chen E, Nguyen J, Thavarajah N, DiGiovanni J, Caporusso F, Chow E (2012). Comparing prognostic factors in patients with spinal metastases: a literature review. Expert Rev Pharmacoecon Outcomes Res.

[52] Titano JJ, Badgeley M, Schefflein J, Pain M, Su A, Cai M, Swinburne N, Zech J, Kim J, Bederson J, Mocco J, Drayer B, Lehar J, Cho S, Costa A, Oermann EK (2018). Automated deep-neural-network surveillance of cranial images for acute neurologic events. Nat Med.

[53] Vargas J, Spiotta A, Chatterjee AR (2018). Initial experiences with artificial neural networks in the detection of computed tomography perfusion deficits. World Neurosurg.

[54] Fabelo H, Halicek M, Ortega S, Shahedi M, Szolna A, Piñeiro JF, Sosa C, O'Shanahan AJ, Bisshopp S, Espino C, Márquez M, Hernández M, Carrera D, Morera J, Callico GM, Sarmiento R, Fei B (2019). Deep learning-based framework for in vivo identification of glioblastoma tumor using hyperspectral images of human brain. Sensors (Basel).

[55] Bae S, An C, Ahn SS, Kim H, Han K, Kim SW, Park JE, Kim HS, Lee S-K (2020). Robust performance of deep learning for distinguishing glioblastoma from single brain metastasis using radiomic features: model development and validation. Sci Rep.

[56] Liu S, Shah Z, Sav A, Russo C, Berkovsky S, Qian Y, Coiera E, Di Ieva A (2020). Isocitrate dehydrogenase (IDH) status prediction in histopathology images of gliomas using deep learning. Sci Rep.

[57] Imani E, Pourreza HR, Banaee T (2015). Fully automated diabetic retinopathy screening using morphological component analysis. Comput Med Imaging Graph.

[58] Lindner L, Narnhofer D, Weber M, Gsaxner C, Kolodziej M, Egger J (2019). Using synthetic training data for deep learning-based GBM segmentation. In 2019 41st Annual International Conference of the IEEE Engineering in Medicine and Biology Society (EMBC). Institute of Electrical and Electronics Engineers..

[59] Ermiş E, Jungo A, Poel R, Blatti-Moreno M, Meier R, Knecht U, Aebersold DM, Fix MK, Manser P, Reyes M, Herrmann E (2020). Fully automated brain resection cavity delineation for radiation target volume definition in glioblastoma patients using deep learning. Radiat Oncol.

[60] Vidotto M, De Momi E, Gazzara M, Mattos LS, Ferrigno G, Moccia S (2019). FCNN-based axon segmentation for convection-enhanced delivery optimization. Int J Comput Assist Radiol Surg.

[61] Altman DG (2009). Prognostic models: a methodological framework and review of models for breast cancer. Cancer Investig.

[62] Moons KGM, Altman DG, Vergouwe Y, Royston P (2009). Prognosis and prognostic research: application and impact of prognostic models in clinical practice. BMJ.

[63] Pesapane F, Volonte C, Codari M, Sardanelli F (2018). Artificial intelligence as a medical device in radiology: ethical and regulatory issues in Europe and the United States. Insights Imaging.

[64] Biganzoli E, Boracchi P, Mariani L, Marubini E (1998). Feed forward neural networks for the analysis of censored survival data: a partial logistic regression approach. Stat Med.

[65] Khan FM, Zubek VB (2008) Support vector regression for censored data (SVRc): a novel tool for survival analysis. Eighth IEEE International Conference on Data Mining, pp 863–868

[66] Vock DM, Wolfson J, Bandyopadhyay S, Adomavicius G, Johnson PE, Vazquez-Benitez G, O'Connor PJ (2016). Adapting machine learning techniques to censored time-to-event health record data: a general-purpose approach using inverse probability of censoring weighting. J Biomed Inform.

[67] Obermeyer Z, Powers B, Vogeli C, Mullainathan S (2019). Dissecting racial bias in an algorithm used to manage the health of populations. Science.

[68] Capper D, Jones DTW, Sill M, Hovestadt V, Schrimpf D, Sturm D, Koelsche C, Sahm F, Chavez L, Reuss DE, Kratz A, Wefers AK, Huang K, Pajtler KW, Schweizer L, Stichel D, Olar A, Engel NW, Lindenberg K, Harter PN, Braczynski AK, Plate KH, Dohmen H, Garvalov BK, Coras R, Hölsken A, Hewer E, Bewerunge-Hudler M, Schick M, Fischer R, Beschorner R, Schittenhelm J, Staszewski O, Wani K, Varlet P, Pages M, Temming P, Lohmann D, Selt F, Witt H, Milde T, Witt O, Aronica E, Giangaspero F, Rushing E, Scheurlen W, Geisenberger C, Rodriguez FJ, Becker A, Preusser M, Haberler C, Bjerkvig R, Cryan J, Farrell M, Deckert M, Hench J, Frank S, Serrano J, Kannan K, Tsirigos A, Brück W, Hofer S, Brehmer S, Seiz-Rosenhagen M, Hänggi D, Hans V, Rozsnoki S, Hansford JR, Kohlhof P, Kristensen BW, Lechner M, Lopes B, Mawrin C, Ketter R, Kulozik A, Khatib Z, Heppner F, Koch A, Jouvet A, Keohane C, Mühleisen H, Mueller W, Pohl U, Prinz M, Benner A, Zapatka M, Gottardo NG, Driever PH, Kramm CM, Müller HL, Rutkowski S, von Hoff K, Frühwald MC, Gnekow A, Fleischhack G, Tippelt S, Calaminus G, Monoranu C-M, Perry A, Jones C, Jacques TS, Radlwimmer B, Gessi M, Pietsch T, Schramm J, Schackert G, Westphal M, Reifenberger G, Wesseling P, Weller M, Collins VP, Blümcke I, Bendszus M, Debus J, Huang A, Jabado N, Northcott PA, Paulus W, Gajjar A, Robinson GW, Taylor MD, Jaunmuktane Z, Ryzhova M, Platten M, Unterberg A, Wick W, Karajannis MA, Mittelbronn M, Acker T, Hartmann C, Aldape K, Schüller U, Buslei R, Lichter P, Kool M, Herold-Mende C, Ellison DW, Hasselblatt M, Snuderl M, Brandner S, Korshunov A, von Deimling A, Pfister SM (2018). DNA methylation-based classification of central nervous system tumours. Nature.

[69] Brat DJ, Aldape K, Colman H, Holland EC, Louis DN, Jenkins RB, Kleinschmidt-DeMasters BK, Perry A, Reifenberger G, Stupp R, von Deimling A, Weller M (2018). cIMPACT-NOW update 3: recommended diagnostic criteria for “Diffuse astrocytic glioma, IDH-wildtype, with molecular features of glioblastoma, WHO grade IV”. Acta Neuropathol.

[70] Chaddad A, Kucharczyk MJ, Daniel P, Sabri S, Jean-Claude BJ, Niazi T, Abdulkarim B (2019). Radiomics in glioblastoma: current status and challenges facing clinical implementation. Front Oncol.

[71] Sperduto PW, Kased N, Roberge D, Xu Z, Shanley R, Luo X, Sneed PK, Chao ST, Weil RJ, Suh J, Bhatt A, Jensen AW, Brown PD, Shih HA, Kirkpatrick J, Gaspar LE, Fiveash JB, Chiang V, Knisely JPS, Sperduto CM, Lin N, Mehta M (2012). Summary report on the graded prognostic assessment: an accurate and facile diagnosis-specific tool to estimate survival for patients with brain metastases. J Clin Oncol.

[72] Kazunari A. (2012), Clinical Evidence for IVD medical devices – Scientific Validity and Performance Evaluation Study Group 5 Final Document GHTF/SG5/N7. Global Harmonization Task Force

