# Supplementary material for: Survival prediction of glioblastoma patients—are we there yet? A systematic review of prognostic modeling for glioblastoma and its clinical potential
Source: Neurosurg Rev. 2020 Nov 6;44(4):2047–57. doi: 10.1007/s10143-020-01430-z (PMC8338817; doi:10.1007/s10143-020-01430-z)
Supplement: Supplementary file 1 — (DOCX 54 kb). [file 10143_2020_1430_MOESM1_ESM.docx]

**Supplementary**

S1 Search Syntax

**Pubmed**

**(**(("Glioblastoma"[Mesh] OR "glioblastoma"[tw] OR "glioblastomas"[tw] OR glioblastom*[tw] OR "grade IV astrocytoma"[tw] OR "grade IV astrocytomas"[tw] OR "grade 4 astrocytoma"[tw] OR "grade 4 astrocytomas"[tw]) AND ("prognostic model"[tw] OR "prognostic models"[tw] OR prognostic model*[tw] OR "prognosis prediction model"[tw] OR "prognosis model"[tw] OR "prognosis models"[tw] OR prognosis model*[tw] OR "prediction model"[tw] OR "prediction models"[tw] OR prediction model*[tw] OR "predictive model"[tw] OR "predictive models"[tw] OR predictive model*[tw])) **OR** (("Glioblastoma"[Mesh] OR "glioblastoma"[tw] OR "glioblastomas"[tw] OR glioblastom*[tw] OR "grade IV astrocytoma"[tw] OR "grade IV astrocytomas"[tw] OR "grade 4 astrocytoma"[tw] OR "grade 4 astrocytomas"[tw]) AND ("Prognosis"[mesh] AND ("Models, Theoretical"[mesh:noexp] OR "Models, Statistical"[mesh]))) **OR** (("Glioblastoma"[majr] OR "glioblastoma"[ti] OR "glioblastomas"[ti] OR glioblastom*[ti] OR "grade IV astrocytoma"[ti] OR "grade IV astrocytomas"[ti] OR "grade 4 astrocytoma"[ti] OR "grade 4 astrocytomas"[ti]) AND (("Prognosis"[mesh] OR "prognosis"[ti] OR prognostic*[ti] OR prognos*[ti] OR "prediction"[ti] OR "predictive"[ti]) AND ("Models, Theoretical"[mesh:noexp] OR "Models, Statistical"[mesh] OR "model"[ti] OR "models"[ti] OR model*[ti])))**)**

**Archivalia**

(("Glioblastoma"[majr] OR "glioblastoma"[ti] OR "glioblastomas"[ti] OR glioblastom*[ti] OR "grade IV astrocytoma"[ti] OR "grade IV astrocytomas"[ti] OR "grade 4 astrocytoma"[ti] OR "grade 4 astrocytomas"[ti]) AND (("Prognosis"[mesh] OR "prognosis"[tw] OR prognostic*[tw] OR prognos*[tw] OR "prediction"[tw] OR "predictive"[tw]) AND ("Models, Theoretical"[mesh] OR "model"[ti] OR "models"[ti] OR model*[ti])) AND ("Mortality"[Mesh] OR "mortality"[Subheading] OR "Survival Analysis"[Mesh] OR "Survival Rate"[Mesh] OR "survival"[tw])) NOT **(**(("Glioblastoma"[Mesh] OR "glioblastoma"[tw] OR "glioblastomas"[tw] OR glioblastom*[tw] OR "grade IV astrocytoma"[tw] OR "grade IV astrocytomas"[tw] OR "grade 4 astrocytoma"[tw] OR "grade 4 astrocytomas"[tw]) AND ("prognostic model"[tw] OR "prognostic models"[tw] OR prognostic model*[tw] OR "prognosis prediction model"[tw] OR "prognosis model"[tw] OR "prognosis models"[tw] OR prognosis model*[tw] OR "prediction model"[tw] OR "prediction models"[tw] OR prediction model*[tw] OR "predictive model"[tw] OR "predictive models"[tw] OR predictive model*[tw])) **OR** (("Glioblastoma"[Mesh] OR "glioblastoma"[tw] OR "glioblastomas"[tw] OR glioblastom*[tw] OR "grade IV astrocytoma"[tw] OR "grade IV astrocytomas"[tw] OR "grade 4 astrocytoma"[tw] OR "grade 4 astrocytomas"[tw]) AND ("Prognosis"[mesh] AND "Models, Theoretical"[mesh])) **OR** (("Glioblastoma"[majr] OR "glioblastoma"[ti] OR "glioblastomas"[ti] OR glioblastom*[ti] OR "grade IV astrocytoma"[ti] OR "grade IV astrocytomas"[ti] OR "grade 4 astrocytoma"[ti] OR "grade 4 astrocytomas"[ti]) AND (("Prognosis"[mesh] OR "prognosis"[ti] OR prognostic*[ti] OR prognos*[ti] OR "prediction"[ti] OR "predictive"[ti]) AND ("Models, Theoretical"[mesh] OR "model"[ti] OR "models"[ti] OR model*[ti])))**)**

**Embase**

(("Glioblastoma"/ OR "glioblastoma".mp OR "glioblastomas".mp OR glioblastom*.mp OR "grade IV astrocytoma".mp OR "grade IV astrocytomas".mp OR "grade 4 astrocytoma".mp OR "grade 4 astrocytomas".mp) AND ("prognostic model".mp OR "prognostic models".mp OR prognostic model*.mp OR "prognosis prediction model".mp OR "prognosis model".mp OR "prognosis models".mp OR prognosis model*.mp OR "prediction model".mp OR "prediction models".mp OR prediction model*.mp OR "predictive model".mp OR "predictive models".mp OR predictive model*.mp))

**Web of Science**

TS=(("Glioblastoma" OR "glioblastoma" OR "glioblastomas" OR glioblastom* OR "grade IV astrocytoma" OR "grade IV astrocytomas" OR "grade 4 astrocytoma" OR "grade 4 astrocytomas") AND ("prognostic model" OR "prognostic models" OR "prognostic model*" OR "prognosis prediction model" OR "prognosis model" OR "prognosis models" OR "prognosis model*" OR "prediction model" OR "prediction models" OR "prediction model*" OR "predictive model" OR "predictive models" OR "predictive model*"))

**Cochrane**

(("Glioblastoma" OR "glioblastoma" OR "glioblastomas" OR glioblastom* OR "grade IV astrocytoma" OR "grade IV astrocytomas" OR "grade 4 astrocytoma" OR "grade 4 astrocytomas") AND ("prognostic model" OR "prognostic models" OR "prognostic model*" OR "prognosis prediction model" OR "prognosis model" OR "prognosis models" OR "prognosis model*" OR "prediction model" OR "prediction models" OR "prediction model*" OR "predictive model" OR "predictive models" OR "predictive model*")):ti,ab,kw

S2 Flowchart of included publications

Domain:

Predictive models for survival in patients with glioblastoma multiforme

Full-text articles excluded
(n = 85)

- Paediatric studies

(n = 10)

- Low grade (I-III) gliomas

(n = 13)

- Extra-axial or secondary tumours

(n = 16)

- Studies focusing on 1 prognostic marker

(n = 37)

- Meeting abstracts

(n = 9)

Studies included in qualitative synthesis
(n = 27)

Full-text articles assessed for eligibility
(n = 112)

Inclusion criteria:

- GBM
- Models focussing on survival
- Clinical models
- Mathematical models
- Models based on machine learning
- English or Dutch

Exclusion criteria:

- Paediatric studies
- Low grade (I-III) gliomas
- Extra-axial or secondary tumours
- Studies focussing on 1 prognostic marker
- Meeting abstracts

Title/abstract screening (n=595)

Records excluded
(n =483)

Records after duplicates removed
(n =595)

Records identified through Pubmed
(n =545)

Additional records identified through Embase, Web of Science, COCHRANE, and Academic search premier (n=50)

## Identification

## Eligibility

## Included

## Screening

S3 Study characteristics of included models

| Study | Source of Data | Types of predictors | Algorithm | Type of  Validation | Model performance |
| --- | --- | --- | --- | --- | --- |
| Youssef et al, 2016 | Registry | Genomics | Cox Prop. | Internal cross | C-index: 0.7+ |
| Michaelsen et al, 2013 | Institutional | Clinical, Genomics | Cox Prop. | Internal cross | C-index:0.82 |
| Peeken et al, 2019 | Institutional | Clinical, Imaging | ML | Internal cross External | C-index (OS): 0.70 |
| Woo et al, 2018 | Institutional | Clinical, Genomics, Imaging | Cox Prop. | Internal cross/External | C-index: 0.70 |
| Liang et al, 2018 | Registry | Clinical, Genomic | Cox Prop. | External | AUC of ROC curve: 0.984 |
| Dehkordi et al, 2017 | Not described | Clinical, pharmacokinetics | ML | k fold cross | C-index: 0.848 |
| Lao et al, 2017 | Registry | Imaging, Clinical | Cox Prop/ML | 10-fold cross | C-index: 0.739 |
| Urup et al, 2016 | Institutional | Clinical | Cox Prop. | External | C-index: 0.58 |
| Park et al, 2017 | Institutional | Clinical, Imaging | Cox Prop. | External | AUC of ROC curve: 0.790 |
| Xia et al, 2017 | Registry | Genomics | CoxSisLasso | Cross | AUC of ROC curve: 0.78 |
| Upadhaya et al, 2015 | Institutional | Imaging | ML | LOOCV | C-index: 0.90 |
| Li et al, 2017 | Registry | Imaging | ML | External | C-index: 0.705 |
| Mazurowski et al, 2013 | Registry | Imaging, Clinical | Cox Prop. | LOOCV | C-index: 0.69 |
| Fuster-Garcia et al, 2018 | Institutional | Imaging, Clinical | Cox Prop. | LOOCV | Accuracy: 0.78 |
| Gittleman et al, 2017 | Clinical trials | Imaging, Clinical, Genomics | Cox Prop. | Bootstrap:  10-fold cross; External | C-index 0.657;  RPA: 0.582 |
| Ai et al, 2017 | Registry | Genomics, Clinical | Cox Prop. | Internal | p = 0.00025 |
| Molitoris et al, 2017 | Institutional | Genomics, Clinical | Cox Prop. | External | C-index: 0.695 |
| Chang et al, 2016 | Institutional | Imaging, Clinical | ML | Internal | HR: 3.64;  accuracy: 0.69 |
| Gorlia et al, 2012 | Clinical trials | Clinical, Imaging | Cox Prop. | Bootstrap | C-index: 0.70; PEV: 15.7% |
| Sanghani et al, 2018 | Registry | Clinical, Imaging | ML | 5-fold cross | Accuracy: 0.87 |
| Audureau et al, 2018 | institutional | Clinical | ML | 10-fold cross External | C-index: 70.37 |
| Yuan et al, 2016 | Institutional | Pathology | Cox Prop. | NA | NA |
| Trister et al, 2013 | Institutional | Imaging | ML | Bootstrap, LOOCV | Bland-Altman |
| Park et al, 2010 | Institutional; database | Clinical, Imaging | Cox Prop. | External | 0.585 |
| Peeken et al, 2018 | Institutional | Clinical, Imaging, Genomics | Cox Prop. | Bootstrap | 0.716 |
| Zacharaki et al, 2012 | Not described | Imaging | ML | Leave 3 out cross | Accuracy: 0.851  AUC:0.84 |

Legend: NA is allocated to the domains that have not been described in the study. LOOCV = Leave out one cross validation.

S4 PROBAST tool used for ROB assessment

| Variables | Qualitative description |
| --- | --- |
| Participants | - 1. Description of data sources were determined from the models.   2. Presence of inclusion and exclusion criteria of participants involved in the development of models. |
| Predictors | - 1. Method of assessing each predictor was determined from the methodology of the models.   2. Whether assessment of predictors was made with knowledge of outcome data was ascertained from the methodology of all models. |
| Outcome | - 1. Overall survival was reported in all models as results. Therefore, a standard outcome definition was used in the included studies.   2. Whether assessment of outcome was made with the knowledge of predictors was ascertained from the results of all models. |
| Analysis | - 1. The number of participants and input data was determined by analysing events per variable from the models.   2. The methodology of each included study stated whether all enrolled participants were included in validating the model. Participants who were excluded had to be handled appropriately.   3. Relevant model performance metrics and performance score was evaluated from the studies.   4. Type of hyperparametric tuning was accounted for the models. |
